# Depolarizing Effects in Hydrogen Bond Energy in 3_10_-Helices Revealed by Quantum Chemical Analysis

**DOI:** 10.3390/ijms23169032

**Published:** 2022-08-12

**Authors:** Hiroko X. Kondo, Haruki Nakamura, Yu Takano

**Affiliations:** 1 School of Regional Innovation and Social Design Engineering, Faculty of Engineering, Kitami Institute of Technology, Kitami 090-8507, Japan; 2 Department of Biomedical Information Sciences, Graduate School of Information Sciences, Hiroshima City University, Hiroshima 731-3194, Japan; 3 Laboratory for Computational Molecular Design, RIKEN Center for Biosystems Dynamics Research, 6-2-3, Furuedai, Suita 565-0874, Japan; 4 Institute for Protein Research, Osaka University, Suita 565-0871, Japan

**Keywords:** 3_10_-helix, hydrogen bond energy, density functional theory, negative fragmentation analysis

## Abstract

Hydrogen-bond (H-bond) energies in 3_10_-helices of short alanine peptides were systematically examined by precise DFT calculations with the negative fragmentation approach (NFA), a modified method based on the molecular tailoring approach. The contribution of each H-bond was evaluated in detail from the 3_10_-helical conformation of total energies (whole helical model, WH_3-10_ model), and the results were compared with the property of H-bond in α-helix from our previous study. The H-bond energies of the WH_3-10_ model exhibited tendencies different from those exhibited by the α-helix in that they depended on the helical position of the relevant H-bond pair. H-bond pairs adjacent to the terminal H-bond pairs were observed to be strongly destabilized. The analysis of electronic structures indicated that structural characteristics cause the destabilization of the H-bond in 3_10_-helices. We also found that the longer the helix length, the more stable the H-bond in the terminal pairs of the WH_3-10_ model, suggesting the action of H-bond cooperativity.

## 1. Introduction

Proteins are macromolecules essential for sustaining life and are also known to perform diverse biochemical functions in nature, such as molecular recognition, chemical catalysis, molecular switching, and the structural maintenance of cells [1,2,3,4]. They typically comprise 20 different amino acids linked by peptide bonds. In aqueous solutions, the polypeptide chains in proteins fold according to their amino acid sequencing and form a three-dimensional structure. The remarkable functional versatility of proteins results from the chemical diversity of the side chains of the constituent amino acids, flexibility of the polypeptide chains, and excellent variety of structures rendered possible by the wide range of amino acid sequences.

Approximately 90% of the amino acid residues in protein structures are found in locally ordered secondary structures, such as an α-helix or a β-sheet [5]. These secondary structures assemble and fold to form three-dimensional structures, also known as tertiary structures. In other words, secondary structures are the building blocks of protein structures.

The helix is the most commonly observed secondary structure and can be classified into different helical conformations [3,4]. Of these, the α-helix is predominant and is found in 80% of the helical structures [6]. The α-helix comprises a remarkably rigid arrangement of polypeptide chains and is a common secondary structural element in fibrous and globular proteins. It is arranged such that the peptide C=O group of the *i*-th residue along the helix faces the peptide N–H group of the (*i* + 4)-th residue, which results in the formation of a hydrogen bond with an N-to-O distance of ~2.8 Å. The second most common helical structure is the 3_10_-helix, which is present in 20% of the helical structures [6]. This helix comprises three amino acid residues per helical turn, and a hydrogen bond is formed between the *i*-th and (*i* + 3)-th residues, resulting in a tighter packing of the backbone compared with the α-helix. This packing also forces the hydrogen bond to move outward from the helical axis.

The hydrogen bond (H-bond) is one of the most important noncovalent interactions for chemical and biological phenomena [7,8,9]. It also plays an essential role in secondary structure formation. Therefore, an accurate and quantitative evaluation of H-bonds is necessary to understand the principles of tertiary structure formation in proteins. It is well known that individual force fields used in classical molecular dynamics (MD) simulations exhibit the specific tendency of generating an α-helix or a β-strand [10,11,12]. Yoda et al. used MD simulations with explicit water molecules to compare the secondary structural properties of commonly used force fields [13,14]. They found that MD simulations using AMBER ff94 [15] and ff99 [16] were in remarkable agreement with experimental data for α-helical polypeptides but not for β-hairpin polypeptides. This preference of force fields on the secondary structure formation is typically not a problem in the MD simulations of rigid globular protein structures. However, it has become a critical issue in understanding functionally critical conformational changes [17,18,19,20] in the folding simulations of flexible disordered regions [17,18] and long loops between secondary structures [19,20]. Numerous attempts have been made to overcome this problem, such as increasing the torsional energies, rearrangements [21,22,23,24], and developing polarized charge models [25,26]. Regardless, the reasons behind the use of these methods remain unclear, and elucidation requires understanding the energy of hydrogen bonding in the secondary structure. 

Several computational chemists have studied hydrogen bonding interactions in secondary structures at various levels of theoretical depth. Wieczorek and Dannenberg investigated H-bond cooperativity and the energetics of α-helices and suggested that various factors contribute to their stability [27,28]. Morozov et al. evaluated the origin of cooperativity in forming α-helices [29]. Zhao and Wu studied the role of cooperativity in the formation of α-helices by performing theoretical calculations on α-helix models constructed using a simple repeating unit method [30]. Parthasarathi et al. studied H-bond interactions in an α-helix model using the atom-in-molecules method [31]. Ismer et al. investigated the temperature dependence of the stability of α-, π-, and 3_10_-helices comparing with a fully extended structure using density functional theory and harmonic approximation [32]. 

In our previous study [33], we calculated the conformational energies of secondary structures formed by alanine oligopeptides using several quantum mechanical (QM) methods; these included the Hartree–Fock (HF) method, second-order Møller–Plesset perturbation theory (MP2), density functional theory (DFT), and molecular mechanics (MM) calculations with classical force-field AMBER ff99SB. The results showed that classical force fields can be used to approximate the energies of parallel and antiparallel β-sheets, which are provided by the QM method. However, the energies of the α-helical structures by the MM method were found to be significantly different from those given by the QM method. This difference might be attributable to the electrostatic energy associated with hydrogen bonding [33]. The molecular tailoring approach (MTA) [34] was slightly modified [35], which is described in detail in Methods and Materials section. This method is denoted here as the negative fragmentation approach (NFA). We used the NFA in combination with DFT and MM calculations to resolve the individual interaction energies associated with each hydrogen bond formed in typical α-helices of different peptide lengths [35]. We concluded that the H-bond energies of the α-helix are generally higher than those of separated H-bonds because of the depolarized electronic structures around the carbonyl oxygen and the participation of amide hydrogen in the H-bond. Such depolarizations redistribute the electron density and are caused by local short-ranged electrostatic interactions with neighboring species in the α-helical structure [35]. 

In the present study, we systematically investigated the H-bond energies and associated electron density changes in 3_10_-helices using QM calculations and compared them with those observed for α-helices in our previous study [35]. The contribution of each H-bond was evaluated from the total conformational energy (whole-helical and WH_3-10_ models). To understand the characteristics of the H-bond energy in 3_10_-helices, we additionally evaluated the H-bond energies using the following simplified models: minimal hydrogen bond (MH_3-10_) models, wherein only H-bond donors and acceptors were present with capping methyl groups, and the single tern (ST_3-10_) model, which includes a single helical turn. The characteristic interactions essential for 3_10_-helices are quantitatively discussed.

## 2. Results 

### 2.1. Structures of H-Bonds of the Optimized Whole-Helical Models of 3_10_-Helices 

We constructed three models of the 3_10_-helices: WH_3-10_, which is oligoalanine peptides capped with the acetyl (Ace) and *N*-methyl amide groups (Nme), referred to as Ace-(Ala)*_n_*-Nme (*n* = 2 to 7) and the simplified models: single turn model (ST_3-10_) and minimal H-bond model (MH_3-10_). We denote WH_3-10_ model of Ace-(Ala)*_n_*-Nme by WH_3-10_-*n* and represent the *s*-th H-bond in WH_3-10_-*n* counting from the N-terminus by *n*-*s*. For comparison, we used the previously reported whole-helical structure models (WH_a_) and simplified models (ST_a_ and MH_a_) of the α-helices. The details of these models are described in Methods and Materials.

**Figure 1:**
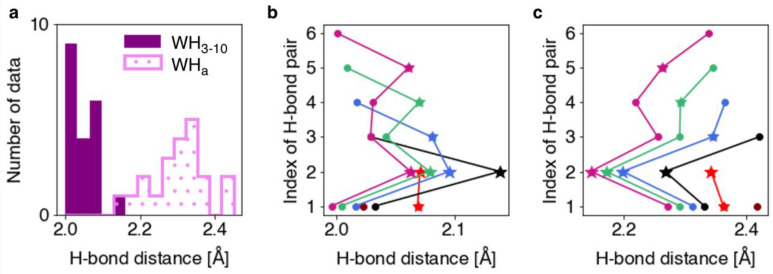
(**a**) Histograms of the H-bond distances in the WH_3-10_ and WH_a_ models. Plots of the H-bond distances in the (**b**) WH_3-10_ and (**c**) WH_a_ models of each length. The H-bond distances of Ace-(Ala)*_n_*-Nme, which forms one-to-six H-bonds, are shown in maroon, red, black, blue, green, and violet–red, respectively. The stars and circles indicate the H-bond pairs adjacent to the terminal pair and the other pairs, respectively.

The H-bond distances and torsion angles were analyzed for the optimized WH_3-10_ models. The H-bond distance was defined as the distance between the oxygen atom in the backbone C=O group and the hydrogen atom in the backbone N–H group, which form an H-bond. A histogram of the H-bond distances and a plot of the H-bond distances for each pair in the WH_3-10_ and the corresponding α-helix models (WH_a_) are shown in Figure 1. The mean values and standard deviations of the H-bond distances for the WH_3-10_ and WH_a_ models were found to be 2.05 ± 0.04 and 2.30 ± 0.07 Å, respectively. The WH_3-10_ models generally exhibited shorter H-bonds than those exhibited by the WH_a_ models. A characteristic feature of WH_3-10_ is that the H-bond distances tend to be greater in the H-bond pairs adjacent to the terminal ones (Figure 1b and Table 1). The 4–2 H-bond pair in WH3-10, sandwiched between the N- and C-terminal H-bond pairs as indicated in the figure of Methods and Materials, showed the longest H-bond distance.

**Figure 2:**
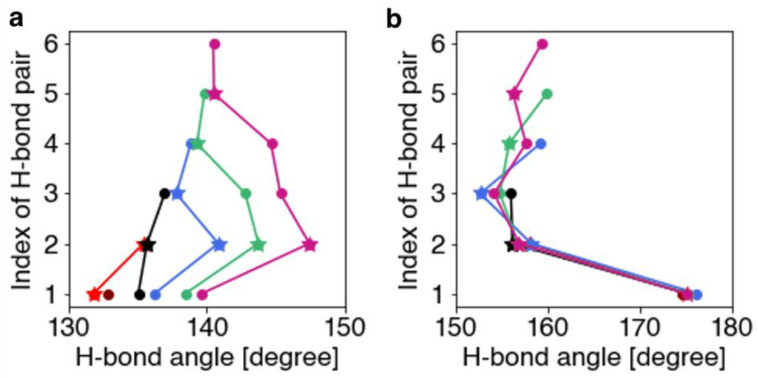
H-bond torsion angles of the (**a**) WH_3-10_ and (**b**) WH_a_ models. H-bond torsion angles of Ace-(Ala)*_n_*-Nme, which forms one-to-six H-bonds, are shown in maroon, red, black, blue, green, and violet–red, respectively. The torsion angles of the H-bond adjacent to the terminal H-bond pair and the other pairs are indicated by stars and circles, respectively.

The H-bond torsion angle was evaluated as the angle between the vectors of the C=O and N–H atoms on the backbone. This corresponds to the dihedral angle of the C, O, N, and H atoms comprising the H-bond. As shown in Figure 2b, the N-terminal H-bonding pairs in the WH_a_ model exhibited significantly different torsion angles than those exhibited by the other H-bonding pairs. In the WH_3-10_ models, the H-bond torsion angles of individual H-bond pairs varied with the length of the helix and their position in it (Figure 2a). In addition, the H-bond torsion angles of the WH_3-10_ models are narrowly distributed, indicating that the molecular backbone of the WH_3-10_ models imposes stronger structural constraints than those of the WH_a_ models. These constraints can possibly lead to considerably shorter H-bond distances in the WH_3-10_ models than in the WH_a_ models, as shown in Figure 1a.

### 2.2. Comparison of H-Bond Energy in Helical Model Systems 

**Figure 3:**
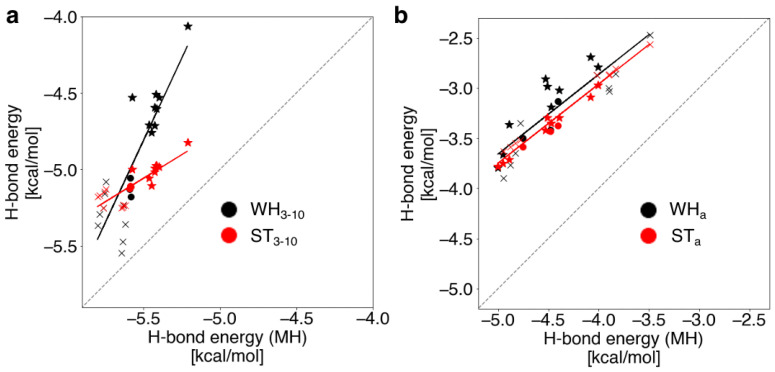
Correlations of the H-bond energies of the (**a**) WH (black) and ST (red) models versus those of the MH model in the 3_10_-helices and (**b**) those in the α-helices [35]. The dashed line shows a guide where the longitudinal axis values have identical H-bond energies. The cross and star marks represent the H-bond pairs of the terminal and those adjacent to the terminal, respectively. The solid lines show regression lines for the H-bond energies of the WH (black) and ST (red) models versus those of the MH model.

To investigate the effects of the helical backbone atoms linking the H-bond acceptor and donor on the H-bond energies, we compared the H-bond energies for the WH_3-10_, ST_3-10_, and MH_3-10_ models of the 3_10_-helices as well as those for the α-helices from our previous study, namely WH_a_, ST_a_, and MH_a_ [35]. In the MH_3-10_ and MH_a_ models, the two peptide groups of hydrogen donors and acceptors were separated without linking the helical backbone atoms. The H-bond energies were calculated with the NFA (Details are shown in Section 4.2). In Figure 3a, the H-bond energies of the WH_3-10_ (black) and ST_3-10_ (red) models are plotted versus those of the MH_3-10_ models for the 3_10_-helices. Those of the WH_a_, ST_a_, and MH_a_ models for α-helices [35] are also shown in Figure 3b.

In the α-helices, the ST_a_ model reproduced the H-bond energies of the WH_a_ model (Figure 3b), indicating that the adjacent residue destabilized the H-bond with respect to the MH_a_ model [35]. In the 3_10_-helices, the ST_3-10_ models also destabilized the H-bond with respect to the MH_3-10_ models, similar to the α-helices, but failed to provide the equivalent H-bond energies. In particular, the H-bond pairs adjacent to the N- or C-terminus were strongly destabilized in energy (underlined in Table S1). This indicates that the helical backbone atoms participating in the H-bond are partly involved in the destabilization of the H-bond but that other factors also lead to unstable H-bond energies. We also found a tendency that the longer the helix length, the more stable the H-bond energy of the N- and C-terminal pairs in the WH_3-10_ model (shown in Table S1). However, there is an exception: The H-bond energies of the N- and C-terminal pairs in WH_3-10_-3 were observed to be higher than those of WH_3-10_-2. They are close to those of the H-bond pairs adjacent to the terminal H-bond pair in the other systems. This would be because the N- and C-termini are adjacent to each other in WH_3-10_-3. For example, the N-terminal H-bond pair of WH_3-10_-3, 3-1, is adjacent to the C-terminal H-bond pair 3-2, and vice versa.

Considering the H-bond energies of the ST_3-10_ model, only the H-bond pairs next to the N- or C-terminal were destabilized by the adjacent residue, as shown in Figure 3a. The pair sandwiched between the N- and C-termini were also the most affected (4-2 of WH_3-10_-4), which causes a difference in the linear regression coefficient between the WH_3-10_ and ST_3-10_ models, as shown in Figure 3a.

To further examine the effect of neighboring residues on the H-bond energy of the 3_10_-helices, we constructed additional models as follows: ST_EC_, in which the ST_3-10_ model, Ace-(Ala)_2_-Nme, was extended to the C-terminal (Ace-(Ala)_2_-Ala-Nme); ST_EN_, in which the ST_3-10_ model was extended to the N-terminal (Ace-Ala-(Ala)_2_-Nme); ST_ECN_, in which the ST_3-10_ model was extended to both N- and C-termini (Ace-Ala-(Ala)_2_-Ala-Nme); and ST_E2N_, in which the ST_3-10_ model was extended by two residues to the N-terminal (Ace-Ala-Ala-(Ala)_2_-Nme). All peptide structures were generated based on the WH_3-10_ model, and their H-bond energies were computed by using the NFA.

**Figure 4:**
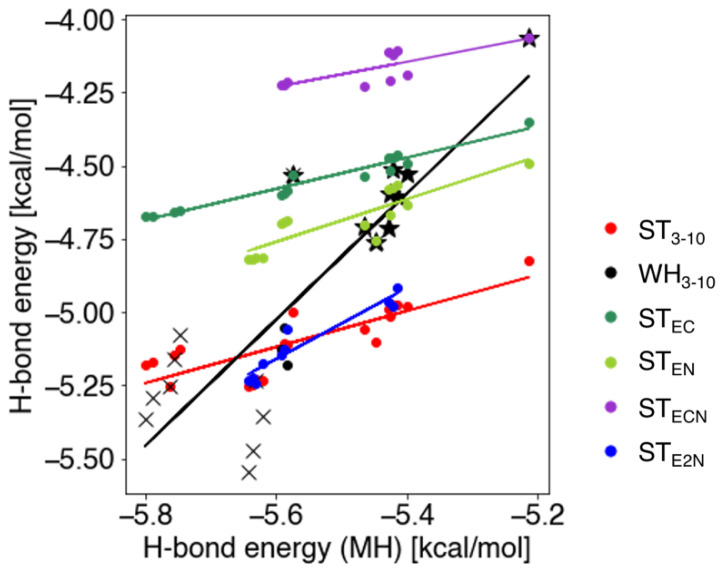
H-bond energies of the extended ST, WH_3-10_, and ST_3-10_ models versus those of the MH model. The cross and star marks of WH_3-10_ represent the H-bond pair of the terminal and that adjacent to the terminal, respectively. The solid lines show regression lines for the H-bond energies of the extended ST, WH_3-10_, and ST_3-10_ models versus those of the MH model.

As expected, in the ST_ECN_ model, both the N- and C-terminal H-bond pairs were adjacent to the target H-bond, thereby making the H-bond energy unstable (Figure 4). The ST_EC_ and ST_EN_ models were more stable than the ST_ECN_ models. The ST_E2N_ model showed nearly the same results as the ST_3-10_ model and higher energy values than those of the terminal pairs in the WH_3-10_ model, as shown in the crosses in Figure 4.

### 2.3. Electronic Structures around the H-Bond Donors and Acceptors

In addition to the H-bond energies, NFA can approximately represent the change of electronic structures upon H-bond formation by Equation (6) in Section 4.2. To examine the difference in the H-bond energy between the ST_3-10_ and MH_3-10_ models and between the WH_3-10_ and ST_3-10_ models in the context of their electronic structures, the differences in the change in electron density were computed using Equations (1) and (2), respectively:

(1) $$\Delta \Delta \rho_{\mathrm{HB}}^{{\mathrm{ST}}_{3-10}-{\mathrm{MH}}_{3-10}}=\Delta \rho_{\mathrm{HB}}^{{\mathrm{ST}}_{3-10}}-\Delta \rho_{HB}^{{\mathrm{MH}}_{3-10}}$$

(2) $$\Delta \Delta \rho_{\mathrm{HB}}^{{\mathrm{WH}}_{3-10}-{\mathrm{ST}}_{3-10}}=\Delta \rho_{\mathrm{HB}}^{{\mathrm{WH}}_{3-10}}-\Delta \rho_{\mathrm{HB}}^{{\mathrm{ST}}_{3-10}}$$

**Figure 5:**
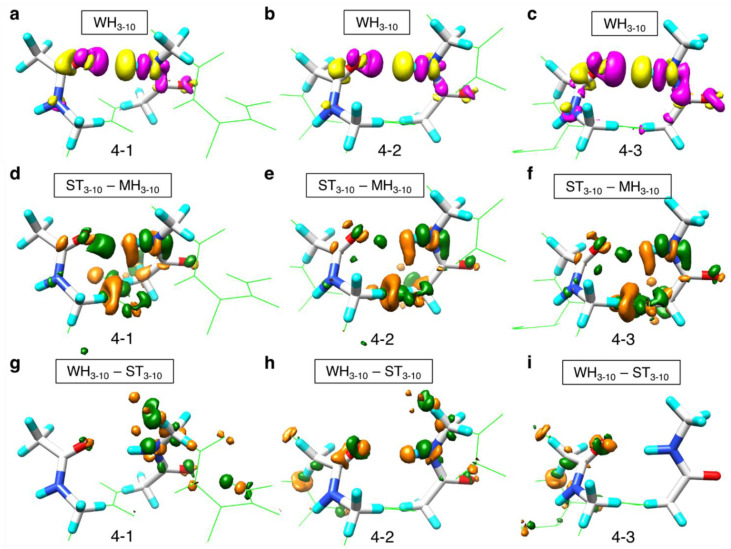
Electron density changes upon H-bond formation,$\;\Delta \rho_{\mathrm{HB}}^{{\mathrm{WH}}_{3-10}}$, for (**a**) 4-1, (**b**) 4-2, and (**c**) 4-3 in WH_3-10_-4. The yellow surfaces represent the contour surfaces at −0.001 au, and the magenta ones are those at 0.001 au. The atoms in the whole WH_3-10_ model are shown by green wire and those in the MH_3-10_ models are shown using the stick model with CPK colors. The difference in the change in electron density between the ST_3-10_ and MH_3-10_ models, $\Delta \Delta \rho_{\mathrm{HB}}^{{\mathrm{ST}}_{3-10}-{\mathrm{MH}}_{3-10}}$, for (**d**) 4-1, (**e**) 4-2, and (**f**) 4-3 in WH_3-10_-4. The difference in the change in electron density between the WH_3-10_ and ST_3-10_ models, $\Delta \Delta \rho_{\mathrm{HB}}^{{\mathrm{WH}}_{3-10}-{\mathrm{ST}}_{3-10}}$, for (**g**) 4-1, (**h**) 4-2, and (**i**) 4-3 in WH_3-10_-4. The dark-green surfaces represent the contour surfaces at –0.00015 au, and the orange ones are those at 0.00015 au.

Figure 5 shows $\Delta \rho_{\mathrm{HB}}^{{\mathrm{WH}}_{3-10}}$ for the first to third H-bond pairs of WH_3-10_-4 (4-1, 4-2, and 4-3), in addition to both $\Delta \Delta \rho_{\mathrm{HB}}^{{\mathrm{ST}}_{3-10}-{\mathrm{MH}}_{3-10}}$ and $\Delta \Delta \rho_{\mathrm{HB}}^{{\mathrm{WH}}_{3-10}-{\mathrm{ST}}_{3-10}}$ for the same pairs. $\Delta \Delta \rho_{\mathrm{HB}}^{{\mathrm{ST}}_{3-10}-{\mathrm{MH}}_{3-10}}$ indicates larger depolarization in the ST_3-10_ model compared with the MH_3-10_ model, as shown in Figure 5d–f. 

As illustrated in Figure 5a–c, we found that the electron density increases in the vicinity of the oxygen atom of the C=O group and that it decreases in the vicinity of the hydrogen atom of the N–H group, thus demonstrating the formation of the H-bond. The $\Delta \rho_{\mathrm{HB}}^{{\mathrm{WH}}_{3-10}}$ of 4-2 appears to be slightly smaller than that of 4-1, indicating a larger depolarization of 4-2 than that of 4-1. This larger depolarization results in a weaker H-bond at 4-2, as illustrated in Figure 5a–c. A remarkable difference was observed between 4-2 and 4-1 and between 4-2 and 4-3: the $\Delta \Delta \rho_{\mathrm{HB}}^{{\mathrm{WH}}_{3-10}-{\mathrm{ST}}_{3-10}}$ of 4-1 and that of 4-3 was considerably smaller than that of 4-2, implying that an effect other than the helical backbone atoms between the acceptor and donor of H-bond pair was at play in the 4-2 pair (Figure 5g,i). Namely, the backbone atoms locating at the N- and C-terminal sides could provide the depolarization effect. This could be the reason behind the difference in the H-bond energy between the WH_3-10_ and ST_3-10_ in the H-bond pairs adjacent to the terminal pairs.

### 2.4. Dependence of Helix Length on H-Bond Energies

**Figure 6:**
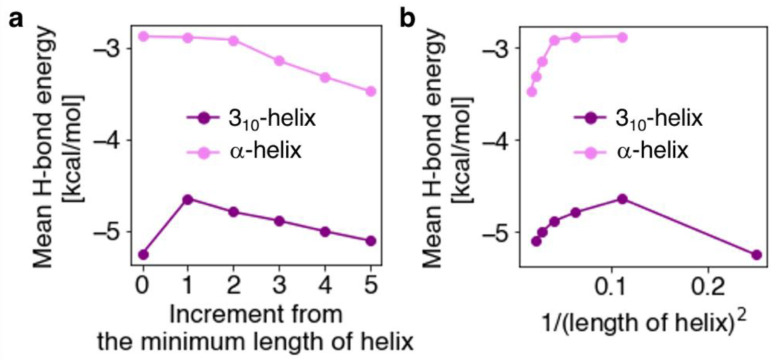
Mean H-bond energies plotted against (**a**) increments of the minimum length of the helix and (**b**) inverse of the square of the helix length in each helical model: *n* = 2 and 3 for the WH_3-10_ and WH_AH_ models, respectively.

We investigated the dependence of helix length on the mean value of the H-bond energies in the WH_3-10_ and WH_a_ models. The mean H-bond energies of these models were plotted as functions of the minimum length of the corresponding helices, as shown in Figure 6. In WH_a_ models, the energy of the H-bond gradually stabilized with an increase in the length of the helix, demonstrating the well-known “H-bond cooperativity” phenomenon [27,28,29,30], where long-range interaction could make more stable helices. The mechanism behind the cooperativity in helix formation can be deconstructed into two parts, namely, electrostatic interactions between residues and nonadditive many-body effects caused by the redistribution of electron density with increasing helix length [29]. In the WH_3-10_ model series, the H-bond of ST_3-10_ was destabilized in the first increment of the minimum length of the helix, while subsequent increments gradually stabilized it. This is because the first increase in helix makes the H-bond adjacent to the terminal H-bond pair, leading to considerable destabilization of the H-bond, as discussed above. Subsequent elongation of the helix results in the stabilization of the terminal H-bond through H-bond cooperativity [28,30].

## 3. Discussion

We systematically investigated the H-bond energies of various 3_10_-helices and found them to exhibit tendencies different from those exhibited by α-helices. Here, we discuss the following three issues: (i) why the H-bond energies in the ST_3-10_ model are destabilized compared with those in the MH_3-10_ model, (ii) why the H-bond pairs adjacent to the terminal pair are largely destabilized compared with other H-bond pairs, and (iii) why the terminal H-bond pairs are stabilized, particularly for long 3_10_-helices.

**Figure 7:**
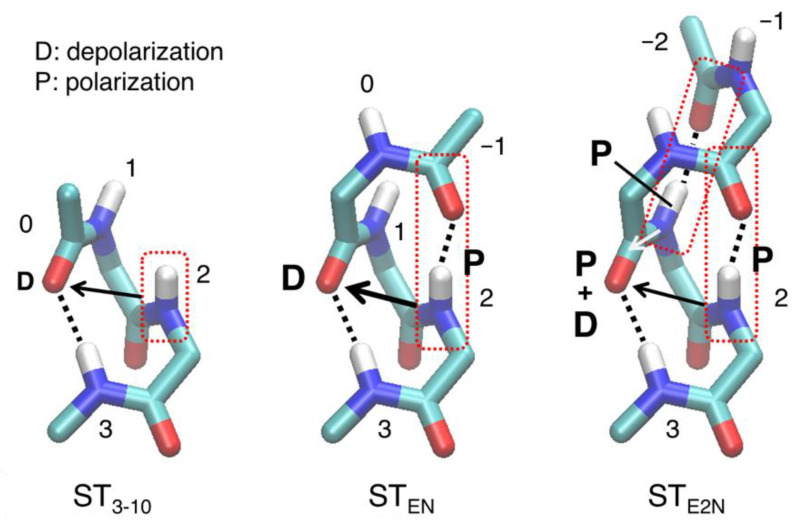
Schematic of the interactions between the H-bond acceptor and neighboring groups. The atoms of the model peptides (ST_3-10_, ST_EN_, and ST_E2N_ models of 7-3 in WH_3-10_ as an example) are shown using the stick model. Hydrogen atoms, except for those in the N–H group, are not shown. The numbers in the figure represent the residue number. Black and white arrows indicate depolarization and polarization effect of the N–H group in the red dotted box on the C=O group in the H-bond, respectively.

For the issue (i), as mentioned in Section 2.3, the C=O and N–H groups participating in the H-bond were depolarized in the ST_3-10_ model in comparison to the MH_3-10_ model. This depolarization could be caused by the helical backbone atoms linking the H-bond pair (residue 2 of Figure 7). In α-helices, the adjacent C=O group is involved in depolarization [35]. However, in the 3_10_-helices, the C=O group of the H-bond pair is closer to the adjacent N–H group (~2.8 Å) than to another adjacent C=O group (~3.4 Å). Therefore, the destabilization of the H-bond is attributed to the depolarization caused by the N–H group.

For the issue (ii), when ST_3-10_ was extended to the N-terminus by a single residue (ST_EN_), an additional H-bond was formed between the C=O group of residue −1 and the N–H group of residue 2. This H-bond formation causes polarization of the N–H group of residue 2, leading to further depolarization of the C=O group of residue −1. Thus, the H-bond could be destabilized. When ST_EN_ is further extended to the N-terminal (ST_E2N_), an additional H-bond is formed between the N=H group of residue 1 and C=O group of residue –2 (back side of the helix in the right panel of Figure 7). This H-bond formation induces polarization of the N–H group of residue 1, causing polarization of the adjacent C=O group of residue 0. This effect could cancel the depolarization effect by the N–H group of residue 2 on the C=O group of residue 0, thereby strengthening the H-bond. At the C-terminal, the similar depolarization effect could destabilize the H-bond.

For the issue (iii), H-bond cooperativity stabilizes the terminal H-bond pair indicated in Section 2.4, as well as the α-helices [27,28,29,30].

For understanding the electronic structures in the more quantitative manner, Hirshfeld population analysis [36,37,38] was performed for WH_3-10_, ST_3-10_, and MH_3-10_ models of 4-1, 4-2, and 4-3, respectively. We calculated the local dipole moments of the C=O and N–H groups of the backbone H-bond acceptor and donor, respectively, as follows [35]:

(3) $${\vec{\mu }}_{\mathrm{CO}}^{i}=\frac{1}{2}\left(q_{C}^{i}-q_{O}^{i}\right)\left({\vec{r}}_{C}^{i}-{\vec{r}}_{O}^{i}\right)$$

(4) $${\vec{\mu }}_{\mathrm{HN}}^{i}=\frac{1}{2}\left(q_{H}^{i}-q_{N}^{i}\right)\left({\vec{r}}_{H}^{i}-{\vec{r}}_{N}^{i}\right)$$

Here, *q^i^*_C_ and *q^i^*_O_ are the Hirshfeld atomic charges of the C and O atoms in the C=O group of the *i*-th residue, and *q^i^*_H_ and *q^i^*_N_ are those of the N–H group of the *i*-th residue, respectively. ${\vec{r}}_{X}^{i}$ is the position vector of the corresponding atom *X* of the *i*-th residue.

The *L*^2^ norm of each dipole moment, $\mu_{\mathrm{CO}}^{i}=∥{\vec{\mu }}_{\mathrm{CO}}^{i}∥$ and $\mu_{\mathrm{HN}}^{i}=∥{\vec{\mu }}_{\mathrm{HN}}^{i}∥$, are used for the following discussion. The resulted $\mu_{\mathrm{CO}}^{i}$ and $\mu_{\mathrm{HN}}^{i+3}$ are shown in Table 2A,B. In the former, the H-bond pairs were formed, and in the latter, the H-bonds were not formed between the C=O group of the *i*-th residue and the N–H group of the (*i* + 3)-th residue.

As clearly shown in Table 2A,B, all Ratio (ST_3-10_/MH_3-10_) and Ratio (WH_3-10_/MH_3-10_) were less than 1. Namely, $\mu_{\mathrm{CO}}^{i}$ and $\mu_{\mathrm{HN}}^{i+3}$ of ST_3-10_ models and those of WH_3-10_ models were always smaller than the corresponding dipole moments of MH_3-10_ models, in which no neighboring carbonyl (C=O) or amide (N–H) groups exist. The amplitude of the depolarization effects relating to the above issue (i) was about 3% to 5% on average from Table 2A,B, independently of whether the H-bonds are formed or not.

Relating to the issue (ii), we found $\mu_{\mathrm{CO}}^{i}$ of WH_3-10_ model of 4-2 was 2% to 3% smaller than those of 4-1 and 4-3, as indicated in Table 2A,B. In contrast, $\mu_{\mathrm{HN}}^{i+3}$ of the WH_3-10_ model of 4-2 had similar values to those of 4-1 and 4-3. These phenomena correlate well with the differences of electron densities shown in Figure 5.

**Figure 8:**
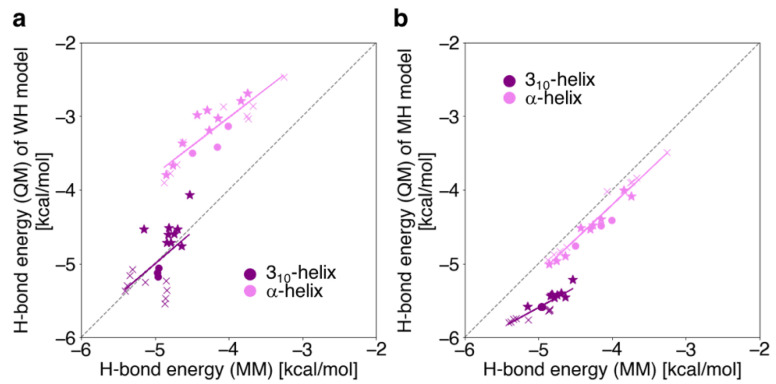
Correlations between the H-bond energies of the (**a**) WH models and the (**b**) MH models, calculated by the QM and MM methods for the 3_10_-helices (purple) and α-helices (violet). The dashed line shows a guide where the H-bond energies of the longitudinal axis values and the MM calculations are identical. The cross and star marks represent the H-bond pair of the terminal and that adjacent to the terminal, respectively. The solid line shows a regression line for each model.

The H-bond energies for the WH_3-10_ and WH_a_ models were compared using QM (*E*_HB_ in Equation (5) in Section 4.2) and MM (*E*_HB_MM_ in Equation (7) in Section 4.2) calculations. Figure 8a exhibits the H-bond energies calculated with QM plotted versus those with MM for individual pairs of the WH_3-10_ and WH_a_ models. In the WH_a_ models, the H-bond energies obtained via QM calculations were shown to be strongly correlated to those obtained via MM, with a correlation coefficient of 0.89. However, the MM calculations overestimated the magnitude of the H-bond energies by ~1 kcal/mol (the mean energy values obtained via QM and MM were −3.21 ± 0.39 and −4.24 ± 0.45 kcal/mol, respectively). Our previous study, wherein the energies were obtained via QM calculation, showed that the destabilization of the H-bond energies in the WH_a_ model was attributed to the depolarization of the H-bond donors and acceptors caused by adjacent residues [35].

In contrast, the H-bond energies for the WH_3-10_ models obtained via QM calculation seem to be closer to those obtained with MM and were more stable than those of the WH_a_ models (the mean energy values obtained via QM and MM for the WH_3-10_ model were −4.96 ± 0.39 and −4.95 ± 0.24 kcal/mol, respectively). Unlike the WH_a_ models, the correlation between the QM and MM calculations was weak: the correlation coefficient was evaluated to be 0.54, as shown in Figure 8a.

In Figure 8b, the H-bond energies calculated with QM are plotted versus those with MM for individual pairs of the MH_3-10_ models and MH_a_ models. Although the H-bond energies obtained via QM and MM for the MH_a_ models almost coincided [35], the H-bond energies obtained by QM were significantly more stable than those obtained via MM for the MH_3-10_ models. However, unlike the WH_3-10_ models, the correlation between the H-bond energies obtained via QM and MM for the MH_3-10_ models was acceptable, and the correlation coefficient was 0.88. The reason why the H-bond energies obtained using MM largely deviate from those obtained via QM may be the poor quality of the atomic partial charges in Equation (7) in Section 4.2. Here, we used the AMBER ff99SB force-field parameters [16] for the atomic partial charges, which were originally determined by multiple-conformation models fitting to the local conformations of a single amino acid for both the α-helical and extended structures [39]. Thus, the parameters may well reproduce H-bond energies for the α-helical conformations MH_a_ but not for the 3_10_-helices MH_3-10_.

In the current study, it is revealed that the H-bond energy of 3_10_-helix largely depends on its local conformation yielding the depolarization and on the long-ranged cooperativity effect. Those QM effects have not been included in the MM computations or for the H-bond energy of the α-helix [35]. In order to improve the MM force fields at least by including the short-ranged interactions, there could be two approaches: (i) by modifying the atomic partial charges, which are not constant values but depend on the local atomic conformation, and (ii) by creating new backbone dihedral parameters, which depends on not only a single amino acid residue but also on the parameter set including the neighboring residues, as suggested by our previous paper [35]. Those approaches may provide us more reliable MM parameters, although they would be difficult to attain.

## 4. Methods and Materials

### 4.1. Whole-Helical Structure (WH_3-10_), Single-Turn (ST_3-10_), and Minimal H-Bond (MH_3-10_) Models

Whole-helical structure models of the 3_10_-helices (WH_3-10_) were constructed using oligoalanine peptides capped with the acetyl (Ace) and *N*-methyl amide groups (Nme), referred to as Ace-(Ala)*_n_*-Nme. We used dipeptide-to-heptapeptide alanines (*n* = 2 to 7) for the WH_3-10_ model, which consists of Ace-(Ala)*_n_*-Nme, is denoted by WH_3-10_-*n*. The backbone dihedral angles (*φ*, *ψ*) for each residue were set to *φ* = −49° and *ψ* = −26°. For comparison, we used the previously reported whole-helical structure models of the α-helices (WH_a_-*n*)*,* composed of the alanine oligopeptide Ace-(Ala)*_n_*-Nme (*n* = 3 to 8), with *φ* = −57° and *ψ* = −47° [3]. These structures were optimized in the gas phase by the energy minimization of the electronic state while keeping the backbone dihedral angles fixed at the aforementioned values. 

**Figure 9:**
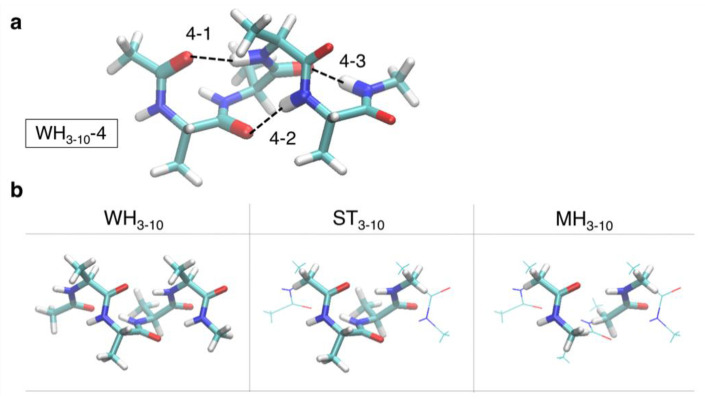
(**a**) Structure of the 3_10_-helix composed of Ace-(Ala)_4_-Nme (WH_3-10_-4) with the notation of each H-bond in the structure. (**b**) Structures of the whole-helical (WH_3-10_), single-turn (ST_3-10_), and minimal H-bond (MH_3-10_) models of WH_3-10_-4, as examples. Carbon, oxygen, nitrogen, and hydrogen atoms are colored in cyan, red, blue, and white, respectively.

One-to-six H-bonds were present between the C=O and N–H groups in the backbone of the optimized WH_3-10_ models, as H-bonds were formed between an *i*-th and (*i* + 3)-th peptide pair for WH_3-10_ and between an *i*-th and (*i* + 4)-th peptide pair for WH_a_. The *s*-th H-bond in Ace-(Ala)*_n_*-Nme, counting from the N-terminus, is represented by *n*-*s* (Figure 9a shows WH_3-10_-4 as an example). The H-bond energies were individually calculated using DFT, as described below.

To analyze the origin of the H-bond interaction energy in 3_10_-helices, we designed two simplified models. One is an ST_3-10_ model, composed of two successive alanine residues capped by Ace and Nme groups at the N- and C-termini, respectively (second column in Figure 9b). The other is an MH_3-10_ model, which comprises two separated *N*-methyl acetamide molecules and mimics a single H-bond between the C=O and N–H groups in the backbone (third column in Figure 9b). The atomic positions of these two models were the same as those of the corresponding WH_3-10_ models, except for the N- and C-terminal capping groups. The computation of the individual H-bond energies for these models was conducted in the same manner as that for each backbone H-bond in the WH_3-10_ models, as described below. The H-bond energies of the three models were then compared.

### 4.2. Calculation of H-Bond Energies Using the Negative Fragmentation Approach

The extraction of the H-bond energy from the total energy of a large molecule wherein the donor and acceptor atoms are linked through several covalent bonds, such as in α- and 3_10_-helices, is not straightforward. In this study, we systematically computed the backbone H-bond energies in the WH_3-10_ models in the same manner as that computing the H-bond energies in the WH_a_ models as reported previously [35], where we modified the MTA developed by Deshmukh et al. [34]. In the NFA, the H-bond energy, $E_{\mathrm{HB}}$, in Ace-(Ala)*_n_*-Nme can be calculated using the following equation:

(5) $$E_{\mathrm{HB}}=E_{\mathrm{sys}}-E_{\bar{A}}-E_{\bar{D}}+E_{\bar{A\cup D}},$$

where $E_{\mathrm{sys}}$, $E_{\bar{A}}$, $E_{\bar{D}}$, and $E_{\bar{A\cup D}}$ are the energies of the entire system, the system lacking the acceptor group, the system without the donor group, and the system lacking both acceptor and donor groups, respectively; detailed descriptions are available in our previous study [35]. In the original MTA, the energy of the entire system was estimated using the energies of all fragments [34]. In the NFA, we used the total energy of the entire system and showed that the difference between the results was negligible [35]. 

The change in the electron density upon H-bond formation, $\Delta \rho_{\mathrm{HB}}$, was evaluated as follows:

(6) $$\Delta \rho_{\mathrm{HB}}=\rho_{\mathrm{sys}}-\rho_{\bar{A}}-\rho_{\bar{D}}+\rho_{\bar{A\cup D}}.$$

For comparison, we also computed the H-bond interaction energies via MM using the AMBER ff99SB force-field parameters [16], $E_{\mathrm{HB}\_\mathrm{MM}}$, for the corresponding H-bonds, as follows:

(7) $$E_{\mathrm{HB}\_\mathrm{MM}}=\sum_{i\in I,j\;\in J\;}\frac{q_{i}q_{j}}{r_{ij}}+\sum_{i\in ,j\in J}\left(\frac{B_{ij}}{r_{ij}^{12}}-\frac{C_{ij}}{r_{ij}^{6}}\right),$$

where *I* and *J* are the four atoms constituting the peptide group, namely, C, O, N, and H, of an acceptor and a donor involved in the H-bond, respectively. *B_ij_* and *C_ij_* are the Lennard–Jones coefficients, *r_ij_* is the distance between the *i*-th and *j*-th atoms, and *q_i_* is the partial atomic charge of the *i*-th atom. The MM energy was calculated for the WH_3-10_ and MH_3-10_ models. 

Calculations for all models were performed using the Gaussian09 program package [40]. The B97D exchange–correlation functional was used with 6–31 + G(d) basis sets. This method is capable of correctly describing van der Waals interactions and is comparable with the MP2 method in the calculation of the H-bond interaction energies of the Ace-(Ala)*_n_*-Nme system in the gas phase [33]. Changes in electron density were computed using cube files provided in the Gaussian09 program packages [40], and molecules that exhibited such changes were depicted by using UCSF Chimera [41]. The other molecular structures were drawn by using the VMD software [42].

## 5. Conclusions

In this study, the H-bond energies and associated changes in the electron density of the atoms forming H-bond of the 3_10_-helices were systematically analyzed using the NFA method with high-quality DFT and MM computations and were compared with those of the α-helices. We prepared optimized structures of Ace-(Ala)*_n_*-Nme, where *n* ranged from 2 to 7 for the whole-helical structure models (WH_3-10_). To quantitatively investigate the origin of the H-bond energy in each helical model, we also constructed single-turn models (ST_3-10_), which comprised two successive alanine residues capped by Ace and Nme groups at the N- and C-termini, respectively, and minimum H-bond models (MH_3-10_), which comprised only pairs of Ace-Nme forming a single H-bond. The structures of the ST_3-10_ and MH_3-10_ models were based on the WH_3-10_ model. The individual H-bond energies were then computed using the NFA.

The distribution of the H-bond distance of the WH_3-10_ models was narrow, and these models exhibited lower values than those exhibited by the α-helical models (WH_a_). The shorter H-bond distance observed in the WH_3-10_ model was due to restrictions imposed by the tight helical structure. The H-bond energy of the WH_3-10_ model exhibited a tendency different from those exhibited by the ST_3-10_ and MH_3-10_ models; it depended on the location of the H-bond pair in the 3_10_-helices. Furthermore, the H-bonds in this model tended to be destabilized in the H-bond pairs adjacent to the terminal pairs and were stabilized at the terminal H-bond pairs. An analysis of changes in the electron density between the WH_3-10_ and ST_3-10_ models and between the ST_3-10_ and MH_3-10_ models suggested that the destabilization of the H-bond in the ST_3-10_ model was attributed to the depolarization caused by the adjacent N–H group. It also suggested that the H-bond formation at this group causes polarization, leading to further depolarization of the C=O group participating in the H-bond pair and larger destabilization of the H-bond adjacent to the terminal H-bond pair. Except for the first increment, the elongation of the helix of the WH_3-10_ model resulted in the stabilization of the terminal H-bond through H-bond cooperativity.

## Figures and Tables

**Table 1:** Mean values and standard deviations of the H-bond distances, *r*_OH_, of the WH_3-10_ and WH_a_ models.

	WH_3-10_^†^	WH_a_
Terminal	2.02 ± 0.02	2.35 ± 0.04
Pairs adjacent to the terminal ^‡^	2.08 ± 0.03	2.26 ± 0.07
Others (inner)	2.03 ± 0.01	2.26 ± 0.03
All	2.05 ± 0.04	2.30 ± 0.07

^†^ H-bond distance is shown in angstroms. ^‡^ The N-terminal H-bond pair in WH_3-10_-3, 3-1, is adjacent to the C-terminal pair, 3-2, and vice versa, and so they are included here.

**Table 2:** Dipole moments of C=O and N–H groups of the backbone H-bond acceptor and donor in in the WH_3-10_-4, ST_3-10_-4, and MH_3-10_-4 models (Debye).

(A) WH_3-10_, ST_3-10_ and MH_3-10_ models where H-Bond pairs exist					
		4-1	4-2	4-3	Average
$\mu_{\mathrm{CO}}^{i}$ ^†^	WH_3-10_ model	1.3523	1.3268	1.3509	1.3433
	ST_3-10_ model	1.3641	1.3877	1.3805	1.3774
	MH_3-10_ model	1.3938	1.4236	1.4139	1.4104
	Ratio (ST_3-10_/MH_3-10_)	0.9787	0.9748	0.9764	0.9766
	Ratio (WH_3-10_/MH_3-10_)	0.9703	0.9321	0.9554	0.9525
$\mu_{\mathrm{HN}}^{i+3}$ ^‡^	WH_3-10_ model	0.5232	0.5386	0.5328	0.5315
	ST_3-10_ model	0.5211	0.5283	0.5293	0.5263
	MH_3-10_ model	0.5371	0.5459	0.5476	0.5436
	Ratio (ST_3-10_/MH_3-10_)	0.9702	0.9678	0.9666	0.9682
	Ratio (WH_3-10_/MH_3-10_)	0.9741	0.9866	0.9730	0.9779
(B) WH_3-10_, ST_3-10_ and MH_3-10_ models in the absence of donor or acceptor					
$\mu_{\mathrm{CO}}^{i}$ ^†^ in the absence of donor (-N–H)	WH_3-10_ model	1.3847	1.3471	1.3883	1.3734
	ST_3-10_ model	1.4026	1.4150	1.4202	1.4126
	MH_3-10_ model	1.4411	1.4594	1.4625	1.4543
	Ratio (ST_3-10_/MH_3-10_)	0.9733	0.9696	0.9711	0.9713
	Ratio (WH_3-10_/MH_3-10_)	0.9609	0.9231	0.9493	0.9443
$\mu_{\mathrm{HN}}^{i+3}$ ^‡^ in the absence of acceptor (-C=O)	WH_3-10_ model	0.5825	0.5873	0.6011	0.5903
	ST_3-10_ model	0.5865	0.5890	0.6003	0.5919
	MH_3-10_ model	0.6194	0.6205	0.6328	0.6242
	Ratio (ST_3-10_/MH_3-10_)	0.9469	0.9493	0.9487	0.9483
	Ratio (WH_3-10_/MH_3-10_)	0.9405	0.9465	0.9500	0.9457

^†^ *L*^2^ norm of dipole moment of the C=O group of *i*-th residue (Debye). ^‡^ *L*^2^ norm of dipole moment of the N–H group of (*i* + 3)-th residue (Debye).

## Data Availability

Not applicable.

## Supplementary Materials

The supporting information can be downloaded from https://www.mdpi.com/article/10.3390/ijms23169032/s1.
