# Supplementary material for: Depolarizing Effects in Hydrogen Bond Energy in 310-Helices Revealed by Quantum Chemical Analysis
Source: Int J Mol Sci. 2022 Aug 12;23(16):9032. doi: 10.3390/ijms23169032 (PMC9409261; doi:10.3390/ijms23169032)
Supplement: Supplementary file 1 [file ijms-23-09032-s001.zip › ijms-1867210-supplementary.pdf]

Depolarizing effects in hydrogen bond energy in 3_{10}-helices revealed by quantum chemical analysis

Hiroko X. Kondo, Haruki Nakamura, and Yu Takano

Table S1. The H-bond energies in the $WH_{3\text{-}10}$, $ST_{3\text{-}10}$, $MH_{3\text{-}10}$, and MM calculations and the H-bond distance of each H-bond pair.

	$WH_{3\text{-}10}$	$ST_{3\text{-}10}$	$MH_{3\text{-}10}$	MM	Distance
**2-1**	-5.253	-5.253	-5.763	-5.136	2.023
**3-1†**	-4.530	-4.998	-5.575	-5.149	2.069
**3-2†**	-4.759	-5.103	-5.447	-4.638	2.071
**4-1**	-5.080	-5.128	-5.747	-5.307	2.033
**4-2**	-4.063	-4.822	-5.213	-4.533	2.139
**4-3**	-5.233	-5.231	-5.630	-4.846	2.029
**5-1**	-5.158	-5.144	-5.755	-5.341	2.016
**5-2**	-4.527	-4.980	-5.399	-4.697	2.096
**5-3**	-4.510	-4.973	-5.420	-4.809	2.081
**5-4**	-5.356	-5.234	-5.619	-4.843	2.017
**6-1**	-5.293	-5.172	-5.788	-5.380	2.004
**6-2**	-4.593	-5.016	-5.426	-4.744	2.079
**6-3**	-5.054	-5.109	-5.586	-4.948	2.042
**6-4**	-4.602	-4.974	-5.414	-4.820	2.070
**6-5**	-5.471	-5.246	-5.635	-4.859	2.009
**7-1**	-5.365	-5.180	-5.801	-5.405	1.996
**7-2**	-4.708	-5.056	-5.465	-4.783	2.063
**7-3**	-5.127	-5.125	-5.59	-4.970	2.029
**7-4**	-5.179	-5.111	-5.582	-4.961	2.031
**7-5**	-4.711	-4.990	-5.428	-4.838	2.061
**7-6**	-5.547	-5.252	-5.642	-4.869	2.001

† The N-terminal H-bond pair in $WH_{3\text{-}10}$-3, 3-1, is adjacent to the C-terminal pair, 3-2, and vice versa.
